# Current Developments of Clinical Sequencing and the Clinical Utility of Polygenic Risk Scores in Inflammatory Diseases

**DOI:** 10.3389/fimmu.2020.577677

**Published:** 2021-01-29

**Authors:** Matthias Hübenthal, Britt-Sabina Löscher, Jeanette Erdmann, Andre Franke, Damian Gola, Inke R. König, Hila Emmert

**Affiliations:** ^1^ Department of Dermatology, Quincke Research Center, University Hospital Schleswig-Holstein, Kiel, Germany; ^2^ Institute of Clinical Molecular Biology, Christian-Albrechts University of Kiel and University Hospital Schleswig-Holstein, Kiel, Germany; ^3^ Institute for Cardiogenetics, University of Lübeck, Lübeck, Germany; ^4^ Institute of Medical Biometry and Statistics, University of Lübeck, Lübeck, Germany

**Keywords:** inflammation, atopic dermatitis, inflammatory bowel disease, coronary artery disease, genome-wide association studies, polygenic risk score, whole-exome sequencing

## Abstract

In this mini-review, we highlight selected research by the Deutsche Forschungsgemeinschaft (DFG) Cluster of Excellence “Precision Medicine in Chronic Inflammation” focusing on clinical sequencing and the clinical utility of polygenic risk scores as well as its implication on precision medicine in the field of the inflammatory diseases inflammatory bowel disease, atopic dermatitis and coronary artery disease. Additionally, we highlight current developments and discuss challenges to be faced in the future. Exemplary, we point to residual challenges in detecting disease-relevant variants resulting from difficulties in the interpretation of candidate variants and their potential interactions. While polygenic risk scores represent promising tools for the stratification of patient groups, currently, polygenic risk scores are not accurate enough for clinical setting. Precision medicine, incorporating additional data from genomics, transcriptomics and proteomics experiments, may enable the identification of distinct disease pathogeneses. In the future, data-intensive biomedical innovation will hopefully lead to improved patient stratification for personalized medicine.

## Introduction

Since sequencing-based high throughput methods have led to cost-effective sequencing of big patient cohorts, our understanding of the genetic background of diseases has evolved. But the more data we are accumulating, the more we understand how complex the genetic background of some diseases is. In chronic inflammatory diseases, such as inflammatory bowel disease (IBD), atopic dermatitis (AD) and coronary artery disease (CAD), research has revealed a number of risk loci that are involved in disease pathophysiology. In spite of the growing number of identified genetic risk genes, functional targeted therapies evolving from our newfound genetic understanding are still in their infancy. The reasons are as manifold as the genetic variants that can lead to complex inflammatory disease. Which variants lead to a phenotype? Which combinations of variations, but not single variants lead to a combined effect that causes physiological impairments? Are patient cohorts where genetic information is derived from predictive for individual patients? And even if we can pinpoint a causative variant, can patients profit from this?

With the rise of high-throughput methods in sequencing we stand on the brink of a revolution in precision medicine. We deepen our understanding of the genetic background that underlies disease on an individual basis and with this we, for the first time, have the tools to implement therapies that distinguish disease subtypes but likewise optimize drug efficacy and minimize side effects. Clinical sequencing for precision medicine can be applied on several levels. Primarily, sequencing provides basic information and with this a characterization of the genetic background of disease. On a second level, genetic information can lead to the generation of prospective knowledge of disease risk, disease severity and disease outcomes. Moreover, sequencing can lead to the identification of subtypes of the disease based on their genetical characteristics.

IBD, AD and CAD represent multifactorial disorders, with genetic as well as environmental factors contributing to the respective clinical phenotype. The complex genetics of these diseases has been comprehensively studied. However, our current understanding of their etiology is still limited. Various studies based on national health registries report an association between the diagnoses of AD and IBD, suggesting a shared pathophysiology. Indeed, e.g., increased TH1/TH17 signaling and the resulting secretion of proinflammatory cytokines represent mutual hallmarks of these diseases (1–3). Likewise, there is evidence for IBD patients to be at an increased risk of atherosclerosis and, consequently, an increased risk for cardiovascular diseases, including CAD. Postulated pathological links between the diseases are manyfold and include the deregulation of inflammatory mediators, dysfunction of endothelial barriers as well as effects of gut microbial endotoxins (4–7).

## High-Throughput Sequencing Provides New Insight on the Genetic Background of Inflammatory Diseases

### Trio Exome Sequencing Reveals Mono- and Oligogenic Forms of IBD

Inflammatory bowel diseases are chronic, relapsing disorders involving inflammation of the gastrointestinal tract caused by the interplay of an overly active immune system and environmental triggers in genetically susceptible individuals. The most common subforms of IBD are Crohn’s disease (CD) and ulcerative colitis (UC). Hundreds of mostly common susceptibility variants have been identified through genome-wide association studies (GWAS), but there are also cases where rare, highly penetrant variants have a large impact on disease. Early-onset cases of IBD (eoIBD), with a disease manifestation during the first 10 years of life, often show a more severe disease course with a higher risk of complications. Furthermore, they are sometimes affected by genetically less complex (monogenic or oligogenic) types of the disease. For example, mutations in genes for the interleukin 10 receptor (IL10R) subunit proteins and the *IL10* gene itself have been shown to be responsible for several cases of severe eoIBD. Recently, we revealed compound-heterozygosity for a missense and a synonymous variant affecting splicing in IL10RA in one patient through trio exome sequencing (8). The *XIAP* (X-linked inhibitor of apoptosis) gene has been shown to be responsible for eoIBD in several male patients. We detected a hemizygous *de novo* nonsense mutation in one of our patients resulting in a selective defect in NOD1/2 signaling (nucleotide-binding oligomerization domain-containing proteins), impaired NOD1/2-mediated activation of NF-κB (nuclear factor “kappa-light-chain-enhancer” of activated B-cells) (9). We also showed a likely synergistic interaction between a rare hemizygous variant in the *NOX1* (NADPH oxidase) gene and a common homozygous variant in the *CYBA* ((Cytochrome B-245 Alpha Chain) gene altering its antibacterial activity in another veoIBD (very-early-onset IBD) patient (10). These examples illustrate the benefit of exome sequencing, and especially trio exome sequencing for diagnostics in eoIBD patients for the identification of *de novo* and compound-heterozygous variants and the reduction of candidate variants in general.

### Association Studies Point to Roles for Common and Rare Variants in AD

Atopic dermatitis is a complex, polygenic, chronic cutaneous disorder. With a lifetime prevalence of up to 20% it represents the most common inflammatory disease of the skin. Atopic dermatitis is believed to be a cutaneous manifestation of a systemic disorder that also gives rise to other atopic conditions, such as asthma and allergic rhinitis. Current models assume a complex interaction between genetic, immunological and environmental factors to be involved in the etiopathogenesis of the disease. For further details, reference is made to recent reviews (11, 12).

A multitude of GWAS has been conducted to detect common variants related to the susceptibility for atopic dermatitis (13–19). In a summarizing meta-analysis Paternoster et al. identified ten novel risk loci, increasing the number of known loci to 31 (20). The most recent association study of rare protein-coding variants incorporating genetic data of as much as 15,574 patients and 377,839 controls resulted in the detection of *DOK2* (docking protein 2) and *CD200R1* (cell surface glycoprotein CD200 receptor 1) as additional susceptibility genes (14). Current estimates of heritability explained by common AD susceptibility variants (minor allele frequency MAF≥1%) amount to 14.91%. An additional 12.56% of heritability are estimated to be attributable to rare protein-coding variants (MAF<1%) (21).

Coding regions for major genes of the late epidermal differentiation have been identified to be colocalized within the so-called epidermal differentiation complex (EDC). Profilaggrin (*FLG*), filaggrin-2 (*FLG2*), and repetin (*RPTN*), represent a subset of EDC gene products contributing to the maturation of the human epidermis. Mutations of the *FLG* gene have been repeatedly shown to be associated with susceptibility and persistence of AD. However, this association could only be observed in individuals of European or Asian ancestry. Based on whole-exome sequencing (WES) mutations of *FLG2* (22, 23), *RPTN* (24), and *CLDN1* (Claudin 1) (25, 26) have been identified to be associated with susceptibility to AD in patients of non-European descent. This suggests factors causing dysfunction of the skin barrier vary across ethnicities (25, 26).

Immunological dysregulation represents another major factor contributing to the etiology of AD. Human leukocyte antigen (HLA) genes, such as *HLA-DRB1* (HLA class II histocompatibility antigen, DRB1 beta chain), play a crucial role for the presentation of antigens to the immune system and have been shown to be associated with the disease. Further immune abnormalities observed in AD and its common comorbidities are caused by mutations of the gene *LRRC32* (Leucine Rich Repeat Containing 32). Using a targeted sequencing approach our group identified and validated low-frequency variants of the gene as strong contributors to AD (27).

### Heritability of CAD and MI Is Only Partially Explained by Currently Known Risk Alleles

Atherosclerotic vascular disease and particularly coronary artery disease remain leading causes of mortality worldwide. Atherosclerosis is initiated by lipid-mediated damage to the endothelium, followed by inflammatory cell recruitment and development of plaques, ultimately leading to plaque erosion or rupture as well as clinical sequelae such as myocardial infarction (MI) or stroke. The use of human genetics to reveal causal mechanisms has proved transformative for deriving aetiological insights in CAD beyond established concepts.

Rare variant analyses have provided examples on how genetic discoveries can point to therapeutic approaches for CAD, e.g., inhibition of HMG-CoA reductase (3-hydroxy-3-methyl-glutaryl-coenzyme A reductase) (28), PCSK9 (proprotein convertase subtilisin/kexin type 9) (29), and ANGPTL4 (angiopoietin-like 4) (30). Genome-wide arrays preferentially contain single-nucleotide polymorphisms (SNPs) that are found at a high frequency in a population as those offer the highest statistical power to detect association. Accordingly, almost all currently identified 164 risk alleles for CAD are common (31). Given the large number and the high frequency of risk alleles that have been identified thus far, virtually every person in our population carries multiple genetic variants that increase susceptibility to coronary disease (32). Each risk allele increases the probability of CAD only by a relatively small margin, i.e., 5–20 relative percentage points per allele. There are two exemptions: One low frequency allele on chromosome 6q25.3 tags markedly increased lipoprotein levels and goes along with a risk increase for coronary disease by 54% (33). The other one is a relatively common variant on chromosome 9p21.3, which increases relative risk by 29% (34).

The rapidly growing list of genetic loci associated with increased risk of CAD is surprising in many aspects. Exemplary, the majority of them has neither been implied in the pathogenesis of the disease (35) nor linked to traditional risk factors (36). Interestingly, the genetic component reflected by common genetic variants cannot explain familial clustering of the disease as well. A positive family history rather appears to be mediated by rare deleterious mutations with a more profound effect (37–39). Not surprisingly, the heritability of CAD and MI is only partially explained by currently known risk alleles (35).

## Clinical Utility of Polygenic Risk Scores

### Association Between Polygenic Risk Scores and Subtypes of IBD

During the past 15 years, GWAS have led to the identification of more than 200 susceptibility loci for inflammatory bowel disease (31, 40). Chen et al. (41) utilized this data to perform a comprehensive comparison of four methods to predict the genetic risk of IBD. With an area under the ROC curve (AUC) of up to 0.78 and 0.70 for CD and UC, respectively, the Bayesian mixture model outperformed the other methods. While this accuracy is not sufficient for diagnostic use in a clinical setting, the authors were able to identify significant associations of higher risk scores with an elevated frequency of bowel resection, earlier disease onset and ileal disease localization. Similarly, Cutler et al. observed a statistically significant relationship between a polygenic liability score and age of onset in pediatric CD patients (42). Ananthakrishnan et al. employed similar methods and found an increasing genetic burden to be associated with earlier age of diagnosis and ileal involvement in CD patients (43). Likewise, a genetic risk score incorporating all known IBD risk alleles showed strong association with disease subphenotypes (44). Predictive models based on this genetic risk score were able to distinguish between colonic and ileal CD. In contrast to adult-onset IBD, veoIBD with an age of onset before the age of six, can be associated with a wide range of rare monogenic, or Mendelian, disorders, but only in a fraction of patients. Serra et al. generated polygenic risk scores based on the effect-size estimates of SNPs significantly associated with adult-onset CD and UC and analyzed whether veoIBD patients with an age of onset under the age of six, harbor a higher load of risk alleles when compared to adult-onset IBD cases or population controls. The risk scores of veoIBD patients were significantly higher compared to those of the healthy controls. However, there was no significant difference between the veoIBD and adult-onset cases (45). In summary, current literature renders polygenic risk scores a promising tool for stratifying IBD patients with regard to age of onset as well as severity of the disease. Current research, including ongoing work of our group, tries to further improve the accuracy of PRS-based predictors to enable future application in a clinical setting.

### PRS-Based Stratification by Disease Susceptibility and Disease Course of AD

Jansen et al. employed additive polygenic risk scores of varying complexity to investigate the putatively increased susceptibility of children diagnosed with cow’s milk allergy (CMA) for common comorbidities, including asthma and AD (46). For AD the authors detected a decreased PRS independent of the employed model. PRS-based prediction of further clinical parameters has been examined in a recent study by Abuabara et al. (47). In populations of varying ethnicity the authors provide evidence for a PRS being highly predictive of AD. However, ancestry-related genetic effects do not independently explain disparities in disease prevalence and disease control between the demographic groups under investigation. Clark et al. investigated the relationship between a PRS and distinct developmental profiles of eczema, wheeze, and rhinitis identified using Bayesian machine learning methods (48). The authors provide evidence for differential association of the PRS across the entirety of developmental profiles, suggesting heterogeneous mechanisms underlying individual disease trajectories. In summary, first studies describe PRS as promising tools for the stratification of cohorts of AD patient with regard to their disease susceptibility and disease course. However, further studies are needed, to replicate these findings.

### Estimating CAD Risk Using PRS

Being a polygenic disease with a substantial heritability, CAD is an attractive target for risk estimation based on the genetic background. Models for risk estimation have already been proposed and entered clinical routine, such as the *HeartScore* (49) and the *Framingham Risk Score* (50). However, these are mainly based on clinical variables. Previously, efforts have been made to improve existing models by the addition of scores based on individual genetic variants (51–55). The PRS used in these approaches were limited by considering only genetic variants for which an association with CAD had previously been established. Recently, this limitation has been abolished by genome-wide polygenic risk scores proposed by Khera et al. (56) and Inouye et al. (57). Using millions of genetic variants to predict the risk of CAD and other complex diseases, these methods outperform model incorporating conventional risk factors. This suggests genetic risk prediction to enable effective prevention strategies.

It is arguable that summarizing the genetic risk using an inherent assumption of linearity is too simple given the complex biological structure of common diseases. Further, estimating the weight of each variant by univariate association tests only neglects possible interactions between variants. Especially in the MHC region, variants can exhibit non-linear effects on diseases through interactions (58–62). To assess this question for CAD, Gola et al. (63) compared various methods from the field of machine learning (ML), which offer attractive algorithms to model non-linear effects, with a GPRS in a case-control data set of samples of European descent from the German population. It turned out that a simple GPRS outperformed all other algorithms under consideration by means of a nested cross-validation. However, the models differed greatly in the number of variants used. While the GPRS utilized ~50,000 variants, the non-linear models were much more sparse, utilizing approximately 1,300 to 10,500 variants. The sheer number of variants in GPRS is an aspect that should not be neglected for their clinical utility: 1. Practical aspects. Although whole genome sequencing becomes cheaper, processing of the data still requires huge computational resources. Traditional genotyping arrays provide a much cheaper and faster way to type variants. However, customary arrays cover about 4.5 million variants, much less than the 6.9 million variants used by Khera et al. 2. Replication. The probability that all variants used by proposed GPRS are available in independent datasets is almost zero. Thus, imputation or proxy variants are necessary, making exact replication of GPRS impossible. 3. Population bias. Using more and more variants to construct GPRS results in overly population specific models. It has already been shown that GPRS developed in individuals of European descent cannot readily be applied to other ethnic groups without taking into account the target population’s structure (64). Yet, it is unknown whether the performance of a GPRS utilising millions of variants depends not only on ethnicity, but also on smaller genomic differences within an ethnicity or even population.

## Clinical Sequencing and Its Implication on Precision Medicine in the Clinical Practice

### Clinical Sequencing Directly Affects Treatment of eoIBD Patients

The most important factors underlying IBD pathogenesis can be summarized as genetics, environment, microbiome, and immunome (indicating the dysregulation of the immune response in the gut) (65). However, the genetic basis alone is extremely complex: The susceptibility variants identified mainly through GWAS explain only a fraction of the expected heritability and most risk loci contain several candidate genes. Only for selected loci, causal variants and genes have been identified in the respective region. The genetic data from GWAS already show associations of some variants or genes with a certain subphenotype and therefore allow the prediction of disease susceptibility and clinical phenotype up to a certain degree. Today, the diagnosis of CD or UC patients still primarily depends on endoscopy or colonoscopy, however, there are also patients in whom CD and UC still cannot be clearly distinguished leading to the unsatisfactory diagnosis of IBD-U (IBD unclassified). Furthermore, IBD patients have an extremely variable disease course, so the expectations for precision medicine do not only include the improvement of diagnostic methods but also the prediction of the disease course and the optimal treatment strategy.

Predictive models based on the genetic risk score were able to distinguish colonic from ileal Crohn’s disease (44). Additional risk scores based on the microbiome may be possible in the future (66) but are not advanced enough as of yet. A transcriptional risk score based on summary-level GWAS and expression quantitative trait locus (eQTL) data integrated with RNA-seq data showed promising results: It outperformed genetic risk scores for discriminating between CD patients and healthy controls and was also able to predict disease course over time in pediatric CD patients (67).

Early-onset forms of IBD highlight the exceptional potential for precision medicine, since the identified variants can be functionally characterized to understand the mechanisms and directly target the disturbed pathways through therapy to correct the consequences of the genetic defect. Thus, in a therapy-refractory IBD patient with a genetic defect in the *XIAP* (X-linked inhibitor of apoptosis) gene and in young children with defects of the IL10 pathway, hematopoetic stem cell transplants (HSCT) were curative (68–71). These examples show the significant progress that has been made towards precision medicine in IBD, but they also highlight the substantial challenges we are facing.

### Clinical Sequencing Enables Monitoring of Established and Development of Novel Treatment Strategies

Traditional treatment approaches for atopic dermatitis include topical as well as systemic therapies. Progress in understanding the pathophysiology of the disease facilitated the development of novel targeted therapeutic options. One classical hallmark of AD is the elevated expression of inflammatory cytokines, including the interleukins IL‐4 and IL‐13, propagating the dysfunction of the epidermal barrier. The monoclonal antibody Dupilumab (Sanofi S.A.), targeting both cytokines represents the first biologic agent approved for the treatment of AD (72). In a recent study Guttman-Yassky et al. provide evidence for the drug progressively improving disease activity, suppressing markers of inflammation and reversing the typical epidermal abnormalities (73). TREATgermany, a non-interventional multicenter patient cohort study has been initiated to assess effectiveness and safety of Dupilumab in the long term. Data collected at follow-up visits of this ongoing study confirm high rates of response without serious side effects (74). Tralokinumab (LEO Pharma A/S) and Lebrikizumab (F. Hoffmann-La Roche AG) represent further emerging treatment options. Tralokinumab, prevents IL‐13 from binding to IL‐13Rα1 as well as IL‐13Rα2 (75). Lebrikizumab, in turn, selectively targets IL-13 and interferes with the formation of the IL-13Rα1/IL-4Rα receptor signaling complex (76). In independent randomized, double-blind, placebo-controlled phase 2b trials in participants with moderate-to-severe AD both drugs showed clear improvements of AD symptoms and an acceptable safety and tolerability profile.

### Genetic Screening for Familial Hypercholesteremia

Large-scale sequencing in a clinical setting is not widely established for CAD, however in case of familial hypercholesteremia (FH) genetic cascade screening is recommended by the World Health Organization (WHO). Untreated FH significantly increases the risk for atherosclerosis and premature CAD (77). Because of its high prevalence and risk of severe complications, it is the only cardiovascular disease recommended for population-based screening by the WHO (78). Current recommendation endorses lipid screening, however genetic testing is encouraged for family-based cascade screening. Genetic testing is also useful to separate heterozygous and homozygous cases, as well as to uncover potential precision medicine targets (77). FH is mainly caused by variants in genes coding for proteins affecting hepatic LDLC uptake including the LDL receptor (*LDLR*), in which most disease-causing variants are found, as well as apolipoprotein B-100 (*APOB*) and proprotein convertase subtilisin/kexin type 9 (*PCSK9*). Multiple studies document the preventive effect of intensive medical LDL-lowering at young age to prevent cardiovascular events (78). Therefore, it has been suggested that incidental detection of variants leading to FH should be communicated to the affected individual and the family (79). In fact, owing to the high frequency of FH, several guidelines recommend programs to systematically unravel variants and to facilitate medical treatment already at young age.

## Perspectives

Clinical sequencing has the potential to reveal directly actionable genetic variants, thus also directly affecting treatment of the patient. For some findings, treatment options already exist, such as HSCT for defects in *XIAP* or the IL10 pathway and monoclonal antibodies for directly targeting imbalanced metabolic processes. Novel findings drive forward the development of new drugs by revealing previously unknown pharmaceutical targets.

Cases reported within this review point to remaining challenges in detecting disease-relevant variants (**Table 1**). For example, the interpretation of synonymous and noncoding variants is still difficult and can lead to false-negative results. A possible complex interplay of rare and common variants, e.g., the two variants in *NOX1* and *CYBA* (3) makes the interpretation of sequencing data even more difficult. Low coverage in genes of interest can lead to reduced detectability of disease-related variants and should therefore be considered with caution. Finally, the choice of public databases represents another crucial factor influencing the results of the analysis. Thus, it needs to be acknowledged that frequency databases may contain (future) patients of the disease under investigation. Databases like ClinVar and HGMD that try to classify variants may also include errors, so variants listed as benign may still be potentially pathogenic, as can be seen for the known Factor-V-Leiden variant that was classified as benign by one submitter in ClinVar. In summary, the greatest bottleneck for clinical sequencing is still the interpretation of data and various factors need to be kept in mind when using NGS data in diagnostics. In the future, deeper sequencing, made possible through further decreasing sequencing costs, novel analysis tools, and the ongoing improvement of variant databases will allow for the more widespread application of clinical sequencing.

**Table 1:** Challenges and possible solutions.

Challenges	Possible solutions
Interpretation of synonymous and non-coding variants, complex interplay of common and rare variants	- Ongoing developments of analysis/prediction tools taking into account findings from large cohort sequencing studies
	- Multi-omics approach combining, e.g., genomic, methylomic, transcriptomic and proteomic data with the microbiome, immunome and exposome
Low coverage of relevant genomic regions	- Deeper sequencing facilitated by a further decrease of sequencing costs
	- Sequencing of exomes or genomes as a replacement for gene panels to include all potentially relevant genomic regions
Large amounts of data from exomes and genomes overwhelm most diagnostic laboratories	- Development of out-of-the-box infrastructural and bioinformatic solutions
Databases, such as ClinVar and HGMD, include questionable variant classifications	- Critical handling and questioning of provided classifications
	- Further expert curation of databases
Risk prediction for complex diseases complicated by heterogeneity of phenotypes and genetic architecture	- Evaluation of impact of model type and complexity on the performance of PRS
	- Ethnicity-specific large cohort studies for evaluation and optimization of PRS performance

This table summarizes challenges of clinical sequencing in the context of inflammatory diseases as identified throughout this review and presents possible solutions.

The shift from gene panels to whole exomes is not yet complete. Exome or whole genome sequencing still poses a challenge for small diagnostic laboratories concerning infrastructural and bioinformatic requirements resulting from the comparatively large volume of generated data. However, the high potential of clinical sequencing is reflected by the increasing rate of solved cases, ending the “diagnostic odyssey” that many patients with rare disorders are facing. Even if the identified genetic cause is not located in a gene that can already be directly targeted in therapy and for which no drugs exist yet, these findings help drive forward the development of future drugs by revealing novel research targets.

**Figure 1:**
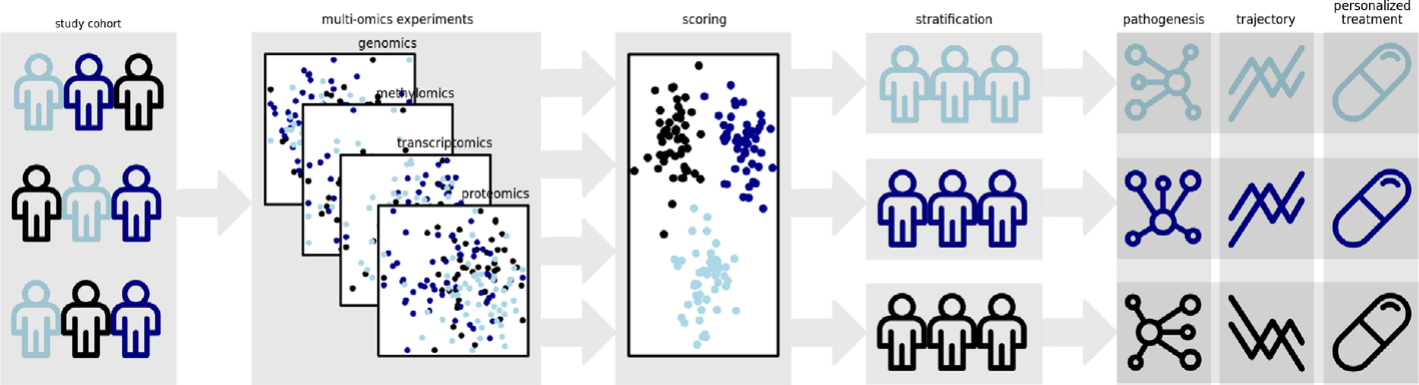
Polygenic risk scores represent promising tools for the stratification of patient groups. Incorporation of additional data from methomics, transcriptomics and proteomics experiments might enable the derivation of multidimensional scoring schemes allowing a more accurate clustering of molecular disease phenotypes. The identification of these disease subtypes might enable the elucidation of distinct disease pathogeneses and trajectories. Ultimately, it will allow custom strategies for care and treatment of the individual patient.

While findings from GWAS and first WES studies lead to the detection of loci being independently associated to the diseases, their interplay has been barely investigated. Furthermore, it can be reasonably assumed that complementing genomic data generated using sequencing technology by other omics layers will help to achieve this objective. Due to their complexity, inflammatory diseases are considered ideal targets for systems biology approaches and integration of multi-omics data. Multi-layered analyses combining, e.g., genomic, epigenomic and transcriptomic data with the microbiome, immunome and exposome are ideally suited to reveal the complex biology underlying the diseases and to identify subphenotypes of the diseases. This knowledge can then be used to develop the ideal treatment, specifically tailored to the patient´s needs and disease characteristics (**Figure 1**).

While the multi-omics approach is a promising strategy, due to its complexity it is not yet feasible to be used in a clinical setting.

Likewise, risk prediction for complex inflammatory diseases is complicated by the heterogeneity of each disease’s phenotype and genetic architecture. Current polygenic risk scores therefore do not yet meet the requirements for diagnosis in the clinical setting. For a number of complex diseases, risk scores are utilized in the stratification of patients in the setting of randomized clinical trials, with the results likely to find their way into clinical practice in the next decade. Thus, in the future, polygenic risk scores may enable patient stratification early on after diagnosis based on their genetic risk and allow for closer monitoring of patients with a high genetic risk that are more prone to stronger disease severity.

## Author Contributions

MH, B-SL, JE, AF, DG, IK, and HE wrote the manuscript. MH, B-SL and HE revised the manuscript critically. All authors contributed to the article and approved the submitted version.

## Funding

This review covers research that has been funded by the Deutsche Forschungsgemeinschaft (DFG) Cluster of Excellence “Precision Medicine in Chronic Inflammation” (PMI, EXC2167).

## Conflict of Interest

The authors declare that the research was conducted in the absence of any commercial or financial relationships that could be construed as a potential conflict of interest.

## Abbreviations

AD, atopic dermatitis; ANGPTL4, angiopoietin-like 4; APOB, apolipoprotein B-100; ATG16L1, autophagy related 16 like 1; AUC, area under the ROC curve; CAD, coronary artery disease; CD, Crohn’s disease; CD200R1, cell surface glycoprotein CD200 receptor 1; DOK2, docking protein 2; EDC, epidermal differentiation complex; eoIBD, early-onset IBD; FH, familial hypercholesteremia; FLG, Profilaggrin; FLG2, filaggrin-2; GPRS, genetic profile risk scores; GWAS, genome-wide association studies; HLA, Human leukocyte antigen; HLA-DRB1, HLA class II, histocompatibility antigen, DRB1 beta chain; HMG-CoA reductase, 3-hydroxy-3-methyl-glutaryl-coenzyme A reductase; HSCT, hematopoetic stem cell transplant; IBD, inflammatory bowel disease; IBD-U, IBD unclassified; IL10R, interleukin 10 receptor; IL23R, interleukin-23 receptor; IRGM, immunity-related GTPase family M; LDLC, low density lipoprotein cholesterol; LDLR, LDL receptor; LRRC32, Leucine Rich Repeat Containing 32; MAF, minor allele frequency; MI, myocardial infarction; NF-κB, nuclear factor ‘kappa-light-chain-enhancer’ of activated B-cells; NOD1/2, nucleotide-binding oligomerization domain-containing protein 1/2; PCSK9, proprotein convertase subtilisin/kexin type 9; PRS, polygenic risk score; RPTN, repetin; SNP, single nucleotide polymorphism; UC, ulcerative colitis; veoIBD, very-early-onset IBD; WES, whole-exome sequencing; WHO, world health organization; XIAP, X-linked inhibitor of apoptosis.
